# Daily Patterns of Preschoolers’ Objectively Measured Step Counts in Six European Countries: Cross-Sectional Results from the ToyBox-Study

**DOI:** 10.3390/ijerph15020291

**Published:** 2018-02-07

**Authors:** Vicky Van Stappen, Delfien Van Dyck, Julie Latomme, Ilse De Bourdeaudhuij, Luis Moreno, Piotr Socha, Violeta Iotova, Berthold Koletzko, Yannis Manios, Odysseas Androutsos, Greet Cardon, Marieke De Craemer

**Affiliations:** 1 Department of Movement and Sports Sciences, Ghent University, Watersportlaan 2, 9000 Ghent, Belgium; Delfien.VanDyck@UGent.be (D.V.D.); julie.latomme@ugent.be (J.L.); Ilse.DeBourdeaudhuij@UGent.be (I.D.B.); greet.cardon@ugent.be (G.C.); marieke.decraemer@ugent.be (M.D.C.); 2 Research Foundation Flanders, Egmontstraat 5, 1000 Brussels, Belgium; 3 GENUD (Growth, Exercise, Drinking Behaviour and Development) Research Group, University of Zaragoza, 50009 Zaragoza, Spain; lmoreno@unizar.es; 4 Children’s Memorial Health Institute, 04730 Warsaw, Poland; P.Socha@IPCZD.PL; 5 Clinic of Paediatric Endocrinology, Medical University Varna, 9000 Varna, Bulgaria; iotova_v@yahoo.com; 6 University of Munich Medical Centre, Dr. von Hauner Children’s Hospital, 80337 Munich, Germany; Berthold.Koletzko@med.uni-muenchen.de; 7 Department of Nutrition and Dietetics, Harokopio University, 80337 Athens, Greece; manios.toybox@hua.gr (Y.M.); oandrou@hua.gr (O.A.)

**Keywords:** preschool, children, physical activity, patterns, hour-by-hour

## Abstract

This study is part of the ToyBox-study, which is conducted in six European countries (Belgium, Bulgaria, Germany, Greece, Poland and Spain), aiming to develop a cost-effective kindergarten-based, family-involved intervention to prevent overweight and obesity in four- to six-year-old preschool children. In the current study, we aimed to examine and compare preschoolers’ step count patterns, across the six European countries. A sample of 3578 preschoolers (mean age: 4.8 ± 0.4) was included. Multilevel analyses were performed to take clustering of measurements into account. Based on the average hourly steps, step count patterns for the six European countries were created for weekdays and weekend days. The step count patterns during weekdays were related to the daily kindergarten schedules. Step count patterns during weekdays showed several significant peaks and troughs (*p* < 0.01) and clearly reflected the kindergartens’ daily schedules, except for Germany. For example, low numbers of steps were observed during afternoon naptimes and high numbers of steps during recess. In Germany, step count patterns did not show clear peaks and troughs, which can be explained by a less structured kindergarten schedule. On weekend days, differences in step count patterns were observed in the absolute number of steps in the afternoon trough and the period in which the evening peak occurred. Differences in step count patterns across the countries can be explained by differences in (school) policy, lifestyle habits, and culture. Therefore, it might be important to respond to these step count patterns and more specifically to tackle the inactive periods during interventions to promote physical activity in preschoolers.

## 1. Introduction

Already in young children, evidence shows a positive relationship between physical activity (PA) and several health outcomes, such as favorable measures of adiposity, bone and skeletal health, motor skill development, psychosocial health, cognitive development and aspects of cardiometabolic health [1,2]. To establish good health in young children and to maintain it later in life, specific PA recommendations for preschoolers (aged 3–5 years) have been developed. The most recent guidelines suggest that preschoolers should be physically active for at least 180 min. at any intensity on a daily basis [1,2], which corresponds to approximately 11,500 steps per day [3].

Across studies, large variation exists in the percentages of preschoolers achieving the PA recommendations. While some studies using accelerometers showed that all preschoolers achieved the recommended 180 min of total PA per day [4,5,6], other research indicated that a high percentage of preschoolers is insufficiently physically active. For example, the results of Hinkley et al. (2012) showed that only 5.1% of Australian preschoolers achieved the recommendation of 180 min per day [7]. Additionally, results of the European ToyBox-study indicated that the number of preschoolers who achieved the PA guideline of 11,500 steps per day across six European countries was between 26.5% (Greece) and 60.7% (Spain) on weekdays and between 20.3% (Greece) and 41.8% (Poland) on weekend days [8]. In addition, in the study of Sigmundova et al. (2016), they found that only 47.4% of the preschool girls and 54.1% of the preschool boys achieved the recommended 11,500 steps per day [9]. These inconsistencies across studies may be due to heterogeneity between samples, differences in monitor type, wear positions and data processing issues, like the cut-point selection [5,10]. Still, the main conclusion was that efforts to promote (moderate-to-vigorous) PA in preschoolers are needed. To find out at which time of the day preschoolers’ PA levels can be enhanced, it is recommended to describe hour-by-hour patterns of preschoolers’ PA during weekdays and weekend days.

Thus far, limited information is available regarding hour-by-hour patterns of preschoolers’ PA levels. Only a few studies previously described PA patterns in preschoolers [5,11,12,13]. In three studies, within-day variability in PA on weekdays and weekend days was examined and showed that less PA-peaks and PA-troughs exist in preschoolers’ PA patterns throughout weekend days compared to weekdays [11,12,13]. During weekend days, comparable results were found across studies, with the lowest PA levels in the morning until 2–3 p.m. [12,13] followed by high PA levels in the afternoon [11,12], followed by a decline in PA levels around 6 p.m. in preschool boys from Denmark (Europe) [11] and in boys and girls from Belgium (Europe) and Melbourne (Australia) [12,13]. In contrast, PA patterns on weekdays differed strongly across the studies. The Belgian study of Verbestel et al. (2011) showed that PA levels from Belgian preschoolers (2.5 to 6 years) on weekdays are related with their daily preschool schedule, showing PA-peaks during recess periods (i.e., morning and afternoon recess and recess during lunch break) and low levels of PA during classroom based activities. The PA pattern of preschool children (3–5 years) in Melbourne showed less peaks and troughs, which can be explained by the fact that data were partly collected during holidays, by a high variability in frequency and duration of preschoolers’ presence at preschool, or a less structured curriculum of Australian preschools [13]. Finally, in the studies of Brasholt et al. (2013) and Hesketh et al. (2015), different peaks and troughs were observed throughout the day. However, in the study of Brasholt et al. (2013), no specific link was described between the daily preschool schedule and preschoolers’ PA-pattern [11]. In the study of Hesketh et al. (2015), it was suggested that the “free-flow” policies in the UK childcare, which allows preschoolers to choose their activities, could account for preschoolers’ high PA-levels during the school day [5]. The results of these previous studies give a general idea of the fluctuations of PA in preschool children. However, based on the different periods of data collection and the fact that the curriculum of preschoolers was not taken into account across the studies, results cannot be compared between the studies.

Based on the study of De Craemer et al. (2015), which used the same cross-sectional data of the ToyBox-study as in the current study, it became clear that the number of preschoolers that achieved the PA-recommendations during weekdays and weekend days differs across European countries (see File 2 from Supplementary Materials: Preschoolers’ PA levels in each country) [8]. However, the paper did not address the differences in detail, so in this study we looked further and investigated PA differences in detail. The creation and description of hour-by-hour step count patterns during weekdays and weekend days across different countries may explain why these differences in daily step counts exist between the European countries. More specifically, hour-by-hour step count patterns will provide more detailed information on preschoolers’ active and inactive time periods during the day.

The aim of the current study was to examine and compare preschoolers’ step count patterns during weekdays (when preschoolers attend preschool) and weekend days, based on hour-by-hour step counts across six European countries participating in the ToyBox-study. Furthermore, the step count patterns during weekdays were related to the country-specific daily kindergarten schedules. We hypothesized that preschoolers’ step count patterns during weekdays and weekend days would differ between the countries and that several school elements (e.g., recess, classroom based activities) would determine the weekday PA patterns across all countries, because of the prearranged kindergarten schedules. A goal of the current study was to try to identify a new window of opportunity to increase preschoolers’ PA during the day across different European countries. Additionally, this study aimed to provide recommendations, which could help to develop more efficient interventions.

## 2. Methods

### 2.1. Sample

For the current study, the baseline measurements of the ToyBox-study were used. The ToyBox-study is a large study conducted in six European countries (Belgium, Bulgaria, Germany, Greece, Poland and Spain), which aimed to develop a cost-effective kindergarten-based, family-involved intervention to prevent overweight and obesity in four- to six-year-old preschool children [14]. Preschoolers were recruited through kindergartens, daycare centers and preschools in the provinces of West- and East-Flanders (Belgium), Varna (Bulgaria), Bavaria (Germany), Attica (Greece), Warsaw and surroundings (Poland) and Zaragoza (Spain). All these settings are referred to as kindergartens in this study. Across all participating countries, kindergarten is not compulsory below the age of five [15] and data on Early Childhood Education and Care in Europe reported that between 74.6% (Poland) and 100% (Spain) of all children between 4 years old and the starting age of compulsory education attend kindergarten [16]. Within each province, lists of existing municipalities were created and information on the socio-economic status (SES) was provided (based on years of education for the population of 25–55 years and/or annual income). Based on the SES information, tertiles (low SES, medium SES and high SES) were created and five municipalities were randomly selected from each tertile. Finally, kindergartens within these municipalities were randomly selected. In total, 309 kindergartens and 7056 children (between 3.5 and 5.5 years old) and their families participated in the study, with a participation rate of 63.3% per kindergarten. Preschoolers’ parents/caregivers were asked to provide written consent before being enrolled in the study. The baseline measurements were conducted between May and June 2012.

Ethical approval was obtained in all six European countries, in line with national regulations (i.e., Ethical Committee of Ghent University Hospital (Belgium), Committee for the Ethics of the Scientific Studies (KENI) at the Medical University of Varna (Bulgaria), Ethikkommission der Medizinischen Fakultät, Ludwig-Maximilians-Universität München (Germany), the Ethics Committee of Harokopio University of Athens (Greece), Ethical Committee of Children’s Memorial Health Institute (Poland), and Comité Ético de Investigación Clinica de Aragón (CEICA) (Spain)).

### 2.2. Measurements

Hour-by-hour step counts were measured by using Omron Walking Style Pro pedometers (HJ-720IT-E2) in Bulgaria, Germany, Greece, Poland and Spain, and Actigraph (GT1M, GT3X and GT3X+ (Pensacola, FL, USA)) accelerometers in Belgium. Accelerometer-based and pedometer-based step counts are valid estimates of preschoolers’ PA and both are comparable (*r* = 0.92) estimates of preschoolers’ PA, which justifies combining pedometer- and accelerometer-based step counts [16]. In Bulgaria, Germany, Greece, Poland and Spain, pedometers were used because of budget restrictions.

Furthermore, preschoolers’ parents/caregivers were asked to fill out the primary caregiver’s questionnaire. Based on this questionnaire, data such as age and sex of the participating child, as well as mothers’ educational level were obtained. In line with Brug et al. (2012), a low educational level was defined as ≤14 years of education, a high educational level as >14 years of education [17]. In European education systems more than 14 years of education implies attendance of higher education (e.g., bachelor program).

### 2.3. Procedure

Preschoolers’ parents/caregivers received an informational letter and were instructed to let their child wear the measurement device (pedometer or accelerometer) for six consecutive days, including two weekend days, and to remove the device during water-based activities and sleeping. The devices were worn at the right hip secured with an elastic belt around the waist. To ensure that preschoolers wore the measurement device correctly, parents received an information letter which contained instructions on how to handle the device. The first (fitting day) and the sixth day (collection day) were omitted from analyses because these two days did not have complete data. Step count data were included if preschoolers had two valid weekdays and two valid weekend days, with a valid day defined as at least 6 h of daily wear time [18]. All daily step counts below 1000 and above 30,000 steps were indicated as missing data [19]. The use of these cutoffs was justified in the study of Rowe et al. (2004) based on the best agreement among three selection methods (e.g., selection method 1: elimination of at least the most extreme 1%). Based on these inclusion criteria, 977 preschoolers were excluded. Pedometer data were downloaded by using Omron Health Management Software version E1.1012 (Omron Health Care Inc., Bannockburn, IL, USA) and accelerometer data were downloaded by using ActiLife version 5.5.5-software (Actigraph, Fort Walton Beach, FL, USA).

### 2.4. Statistical Methods

Descriptive analyses were executed using SPSS statistics version 22 (SPSS Inc., Chicago, IL, USA) and were reported as means and standard deviations (SD). An independent samples *T*-test was conducted to compare the average daily step counts between preschool boys and girls, separately for weekdays and weekend days. Additionally, multilevel repeated measures analyses were performed using MLwiN 2.32 to explore hour-by-hour step count patterns for weekdays and weekend days across the six European countries. MLwiN is a commonly used software package for fitting multilevel models into Windows [8,20,21] (http://www.bristol.ac.uk/cmm/software/mlwin/). Average hourly step counts were calculated between 6 a.m. and 11 p.m. and the averages per hour were compared with each other (e.g., average step counts at 6 a.m. was compared with average step counts at 7 a.m., etc.). In line with the study of Collings et al. (2013), periods before 6 a.m. and after 11 p.m. were not included in the analyses because no step counts were recorded in the majority of preschoolers and because this period also reflected their sleeping time [6]. To take clustering of measurements into account, four-level multilevel analyses were performed (time, child, classroom, and kindergarten). All analyses were adjusted for sex, age and mothers’ educational level. Statistical significance level was set at *p* < 0.01 to account for multiple testing.

**Figure 1:**
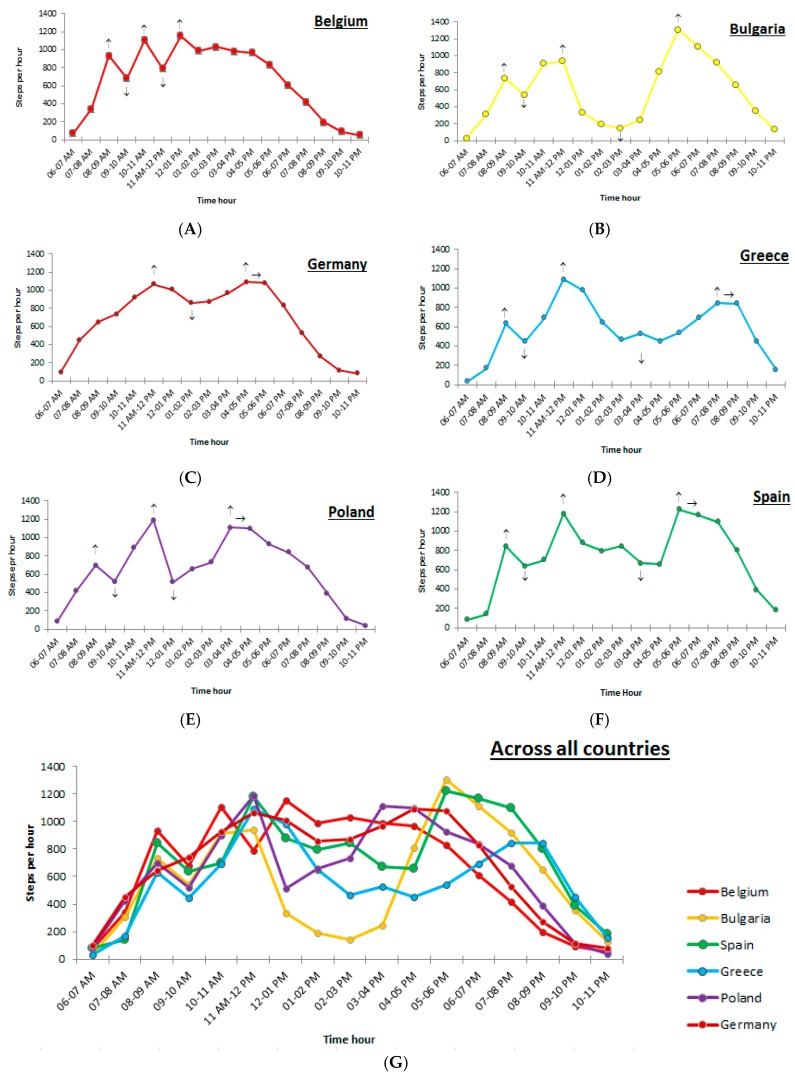
(**A**) Preschoolers’ step count patterns during weekdays in Belgium; (**B**) preschoolers’ step count patterns during weekdays in Bulgaria; (**C**) preschoolers’ step count patterns during weekdays in Germany; (**D**) preschoolers’ step count patterns during weekdays in Greece; (**E**) preschoolers’ step count patterns during weekdays in Poland; (**F**) preschoolers’ step count patterns during weekdays in Spain; and (**G**) preschoolers’ step count patterns during weekdays across all participating countries.

**Figure 2:**
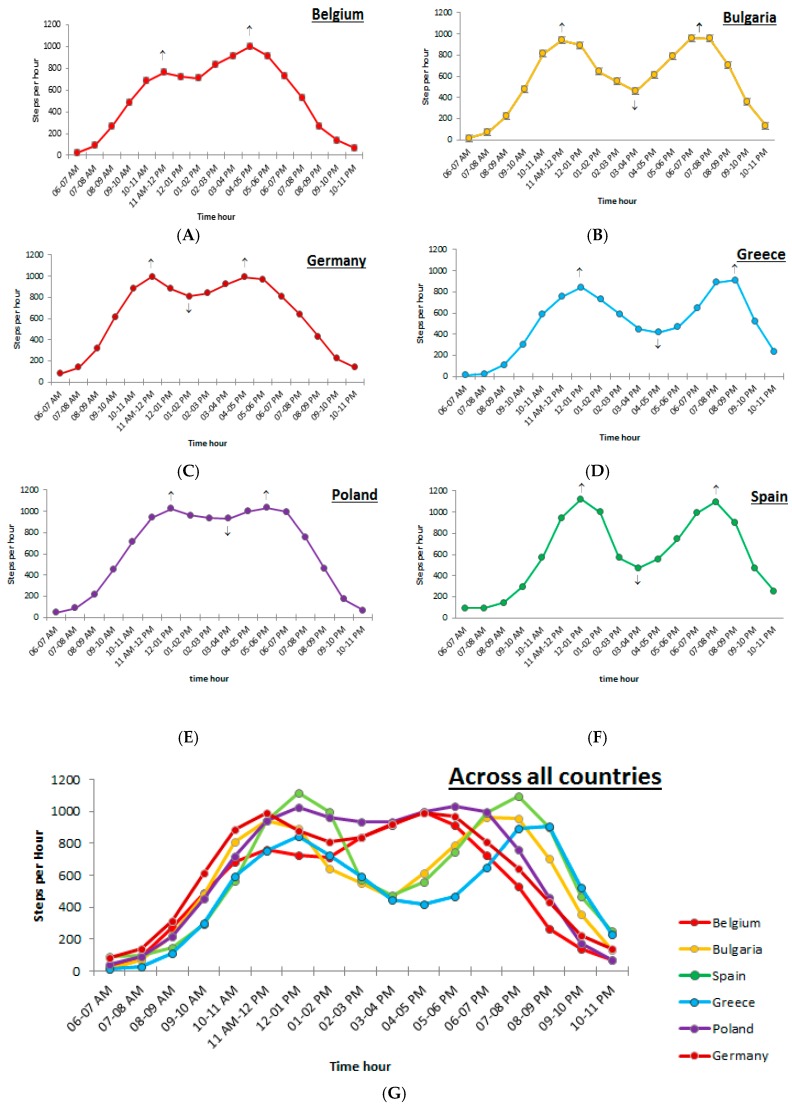
(**A**) Preschoolers’ step count patterns during weekend days in Belgium; (**B**) preschoolers’ step count patterns during weekend days in Bulgaria; (**C**) preschoolers’ step count patterns during weekend days in Germany; (**D**) preschoolers’ step count patterns during weekend days in Greece; (**E**) preschoolers’ step count patterns during weekend days in Poland; (**F**) preschoolers’ step count patterns during weekend days in Spain; and (**G**) preschoolers’ step count patterns during weekend days across all participating countries.

Based on the average hourly step counts, step count patterns were created for the six European countries, separately for weekdays and weekend days. To describe the fluctuations throughout the day, significant peaks and troughs were indicated. A significant peak in step counts occurred when a significant increase in step counts (*p* < 0.01) was observed, followed by a significant decrease in step counts (*p* < 0.01) (indicated on the step count patterns (Figure 1 and Figure 2) by “↑”). When a significant increase in step counts (*p* < 0.01) occurred, followed by a stagnation in step counts (i.e., no significant increase or decrease, *p* < 0.01) this was described as a plateau-peak (indicated on the step count patterns (Figure 1 and Figure 2) by “→“). Finally, a significant trough occurred when a significant decrease (*p* < 0.01) was followed by a significant increase in preschoolers’ step counts (*p* < 0.01) (indicated on the step count patterns (Figure 1 and Figure 2) by “↓”).

All significant peaks and troughs during weekdays were related to the daily schedules from the kindergartens per country. In File 1 from Supplementary Materials, the country-specific daily kindergarten schedules are described.

## 3. Results

In this cross-sectional study, valid data were obtained from 11,500 preschoolers (52.3% boys) between 3.5 and 5.5 years old with a mean age of 4.8 (±0.4) years. Descriptive statistics of the sample, reported per country, can be found in Table 1. Differences in daily step counts were observed between preschool boys and girls, both on weekdays and on weekend days (respectively: *t* = 11.265, *p* < 0.01 and *t* = 6.95, *p* < 0.01). On weekdays and weekend days, preschool boys achieved a higher amount of step counts compared to preschool girls. The differences in preschoolers’ daily step counts are shown in Table 2.

### 3.1. Step Count Patterns during Weekdays

#### Description of Significant Peaks and Troughs Related to Daily Kindergarten Schedules

During weekdays, preschoolers’ step count patterns showed different significant peaks and troughs in step counts across all countries (Figure 1A–G). The number of step counts in all significant peaks and troughs can be found in Table 3. A distinction was made between general peaks and troughs (i.e., peaks and troughs that could be found across at least five countries) and peaks and troughs in the six European countries. In the section below, only the general peaks and troughs will be presented. The step count pattern during weekdays will be related to kindergartens’ daily schedules across the different countries (see File 1 from Supplementary Materials).

In total, three significant general peaks were observed. The first significant general peak (*p* < 0.01) occurred between 8 and 9 a.m. in all countries, except for Germany where no peak could be observed. Between 8 and 9 a.m. was the starting time of the kindergartens in these countries. In this first peak, step counts from 632 (Greece) to 931 (Belgium) steps per hour were observed. The second significant general peak (*p* < 0.01) was found in all countries and can be related to a recess period, just before (Bulgaria, Spain, Poland, Germany and Greece: 11–12 p.m.) or after lunchtime (Belgium: 12–1 p.m.). In this general peak around 12 p.m., step counts from 940 (Bulgaria) to 1188 (Poland) were observed. Finally, a third general significant peak (*p* < 0.01) was observed during the late afternoon and evening in all countries, except for Belgium. In this third general peak, preschoolers’ step counts raised up to 529 (Greece)–1306 (Bulgaria) steps per hour. The timing of this third peak clearly differed between countries. In Germany, Greece and Poland, this peak took place at kindergartens’ closing time and when preschool children went home (Poland: between 3 and 4 p.m.; Germany: between 4 and 5 p.m.). In Bulgaria and Spain, this last significant general peak occurred during after school hours, between 5 and 6 p.m. In Germany, Greece, Poland and Spain, the last peak was maintained for two hours and is therefore called a plateau-peak. In the step count patterns from Belgian preschoolers no third significant general peak was observed, however the step count pattern showed a relatively high number of steps for two hours after Belgian kindergartens’ closing time (3 p.m.).

Furthermore, two significant general troughs were observed during weekdays and were also related to the daily schedule of preschool children. The first significant general trough (*p* < 0.01) occurred between 9 and 10 a.m., when classroom based activities (Belgium, Greece, Poland and Spain) or breakfast (in Bulgaria) were scheduled. Only in the German step count pattern, no significant trough was observed in this period. In this first trough, preschoolers’ step counts decreased to 523 (Poland)–681 (Belgium) steps per hour. The second significant general trough (*p* < 0.01) was observed in the afternoon (between 12 and 4 p.m.), when preschoolers have an afternoon sleep period (Germany, Greece, Bulgaria, and Poland), and/or classroom based activities (Spain, Germany, and Greece). Step counts from 145 (Bulgaria) to 670 (Spain) steps per hour were observed. Only in Belgium, no significant trough was observed in this period.

### 3.2. Step Count Patterns during Weekend Days

#### Description of Significant Peaks and Troughs

During weekend days, comparable step counts-patterns were observed across the different countries (Figure 2A–G), except for Belgium. In Bulgaria, Germany, Greece, Poland and Spain, a first significant peak occurred, followed by a significant trough, followed by a significant second and final peak. However, differences exist in the number of step counts in peaks and troughs and the period in which peaks and troughs occurred across the different countries. The step counts in the first and second peaks as well as the step counts in the trough can be found in Table 4. In Belgium, two significant increases in step counts (*p* < 0.01) were observed (one on the morning and one in the evening), however, no significant trough was observed.

Between 11 a.m. and 1 p.m., the first significant peak occurred in the countries (*p* < 0.01), except for Belgium. This peak was observed between 11 a.m. and 12 p.m. in Bulgaria and Germany, and between 12 and 1 p.m. in Greece, Poland and Spain. Preschoolers reached step counts from 842 (Greece) to 1119 (Spain) steps per hour. A second significant peak occurred between 4 and 9 p.m. (*p* < 0.01), when preschoolers’ number of step counts increased up to 909 (Greece)–1093 (Spain) steps per hour. More specifically, a high number of step counts was observed between 4 and 5 p.m. in Germany, between 5 and 6 p.m. in Poland, between 6 and 7 p.m. in Bulgaria, between 7 and 8 p.m. in Spain and between 8 and 9 p.m. in Greece. Finally, in the afternoon, a significant trough (*p* < 0.01) was observed between 1 and 5 p.m. across all countries, except for Belgium. The timing of this trough clearly differed between countries, namely the lowest number of steps in this trough was found between 1 and 2 p.m. in Germany; between 3 and 4 p.m. in Bulgaria, Spain and Poland; and between 4 and 5 p.m. in Greece. Across the participating countries, the number of step counts in the trough varied between 417 (Greece) and 933 (Poland) steps.

## 4. Discussion

When comparing the step count patterns across the six European countries, during weekdays, several peaks and troughs were observed and step count patterns clearly differed across the countries. However, when taking into account the country-specific kindergarten schedules (see File 1 from Supplementary Materials), it is clear that kindergartens’ scheduling (e.g., recess, classroom based activities, and afternoon sleep period) strongly reflected the number of steps in preschool children during kindergarten hours. German preschoolers’ step count pattern showed less significant peaks and troughs during weekdays in comparison to the other participating countries. This can be explained by the fact that German kindergartens can schedule their activities (e.g., recess, lunch break, and afternoon sleep periods) individually [22], which means that the daily kindergarten schedules differ across the kindergartens in Germany.

A previous study, which also used the cross-sectional data of the ToyBox-study, investigated preschoolers’ daily step counts during weekdays and showed that the number of preschoolers that achieve the PA guidelines of 11,500 steps per day [3] differs between countries (see File 2 from Supplementary Materials) [8]. De Craemer et al. (2015) found that, in Spain, the highest percentage of preschool children achieved the recommended 11,500 steps per day, namely 60.7%, followed by Germany, where 49.9% of the preschoolers achieved the PA recommendations. In the current study, the step count pattern in Spain is characterized by several peaks and troughs throughout the day and higher step counts for about 2 h in the evening. This short retention period of high steps for about 2 h may partly result in a high percentage of preschoolers that achieved the daily step count recommendation in the study of De Craemer et al. (2015) [8]. In Spain, a number of successful regional and local initiatives exist to encourage after-school physical activities [23]. Thus, the availability of these initiatives could be a possible reason for the short retention period of Spanish preschoolers’ high step counts in the evening. However, to the best of our knowledge, no data are available on the amount of preschoolers that participate in these extra-curricular activities. In addition, in the other Southern European countries (i.e., Bulgaria and Greece), higher levels of step counts were observed during after school hours compared to the Central European countries (i.e., Belgium, Germany and Poland). This might be explained by better weather conditions in the Southern countries (i.e., Bulgaria, Greece and Spain), and the fact that Southern European people also have different lifestyles, staying outside for a longer period of time in the evening, compared to Central European people. A possible effective strategy to achieve higher number of step counts during after school hours across all countries could be to stimulate both structured and unstructured PA [12]. Kindergartens could be encouraged to organize extracurricular activities, during after school hours, in the kindergarten setting [24] and in the home environment, preschool children can be encouraged to play actively at home or families can be stimulated to be active with their preschool children. Additionally, it seems to be important to stimulate outdoor play as results showed that time spent outdoors is positively associated with children’s PA (aged 3–12 years) [25].

Next to Spanish preschoolers, a relatively high number of German preschoolers also achieved the recommended step counts during a weekday (i.e., 49.9%) [8]. Based on this finding, the approach used in Germany (i.e., kindergartens that can schedule their program individually) may be effective to reach a higher number of preschoolers achieving the PA recommendations. This is in line with the findings of the study of Hesketh et al. (2015), who showed that all preschoolers in Cambridgeshire (the UK) achieved the PA guideline of 180 min. per day, which may be due to the kindergarten “free-flow” policy allowing preschoolers to choose their activities, freely moving between inside and outside environment for most of the day [5]. In the other countries, clear step count patterns were observed, which could mean that preschoolers’ schedules are more fixed in these countries. This shows that preschoolers are almost only able to play at pre-arranged time periods (i.e., during recess), which is comparable with the findings of PA patterns in primary school aged children [26]. In Germany, this kindergarten-specific approach may provide more flexibility to teachers to meet the needs of the preschoolers, which may result in more PA during kindergarten hours and consequently, in a higher amount of preschoolers that reach the PA recommendations.

As most troughs in step counts during school hours were probably caused by classroom based activities, this might suggest that those activities are not activity enhancing. Similar results were found in the study of Verbestel et al. (2011), who indicated that blocks of sedentary classroom based activities are already present in Flemish preschool children [12]. To enhance preschoolers’ PA during school hours, it can be recommended for teachers to integrate more PA (i.e., implement PA in the daily curriculum or introduce movement breaks) into their daily classroom based activities. Trost, Fees and Dzewaltowski (2008) concluded that integrating PA in all aspects of the kindergarten curriculum (e.g., into mathematics, nutrition education) is feasible and a potential effective strategy to increase PA in preschool children [27]. In addition, incorporating classroom based PA-breaks seemed to be effective to increase preschoolers’ PA levels during the school day [28].

Across all countries, relatively high numbers of step counts were found during recess periods during the hours at kindergarten (i.e., at kindergartens’ starting time, during the recess period just before or after lunch time and during kindergartens’ closing time). Similar results were found in the study of Verbestel et al. (2011) in which preschoolers’ recess period was associated with the highest PA levels during the day [12]. In the study of Cardon et al. (2008), results showed that preschoolers’ higher step counts per minute were significantly associated with more space per child and with shorter recess time. Therefore, it is recommended for teachers and headmasters to provide sufficient space during recess, and if necessary to split into groups with different recess times [29].

The lowest numbers of preschoolers that achieved the PA recommendations on weekdays were found in Bulgaria and Greece, respectively, 29.3% and 26.5% [8]. In Bulgaria, this result may be partly explained by the afternoon sleep period that is scheduled in the kindergarten program (between 1 and 4 p.m.). Remarkably, all preschools in Bulgaria are equipped with beds for afternoon naptimes for each preschool child [30]. In addition, in some other countries, namely Germany, Greece, Poland and Spain, preschoolers get the opportunity to nap in the afternoon (see File 1 from Supplementary Materials). Giving the opportunity for a nap may benefit some preschoolers, particularly those who are younger and had less than 10 h of sleep the previous night [31]. However, for other preschoolers or preschool children that had sufficient hours of sleep during the night, restlessness, anxiety and aggression during and after the afternoon rest period may occur [31]. Therefore, we recommend that kindergartens avoid systematically putting all preschool children in bed in the afternoon and, instead, respond to the needs of each individual preschooler.

During weekend days, a relatively high proportion of Polish and Spanish preschoolers achieved the daily PA recommendations of 11,500 steps (respectively 41.8% and 37.0%) [8]. However, this study showed that the step count patterns of Poland and Spain have different fluctuations in step counts over time. These step count patterns differ at two important time points. More specifically, the afternoon trough in Spain is deeper (i.e., lower step counts) compared to Poland, and the late-evening peak (last peak) is higher in Spain compared to Poland. The fluctuation of the PA pattern in Spain (i.e., a deep afternoon trough and late-evening peak) was also found in the other Southern European countries (i.e., Bulgaria and Greece) which might be due to the siesta which takes place during the warmest part of the day. The late-evening peak might be explained by Southern European children staying active for a longer period during the day due to hotter weather conditions, compared to the Central European countries. A possible strategy to promote PA in preschoolers could be to take into account the above-mentioned differences in step count patterns. In Southern European countries, not ignoring the importance of taking a siesta and the hot weather conditions, a possible strategy could be to promote PA, especially in the morning and in the late afternoon or evening. Furthermore, it might be an option to shorten the afternoon trough by promoting PA in more comfortable temperatures (e.g., indoor PA). As the afternoon trough cannot really be explained in the Central European countries, weekend afternoons are a good opportunity to enhance preschoolers PA levels.

A limitation of this study was the use of PA patterns based on step counts instead of accelerations. Due to the high cost of accelerometers (~US $249 or €190 per device) it was not possible to use these devices in all countries (except for Belgium). Based on the literature, it is clear that pedometers are less accurate to measure PA, as they cannot measure PA intensities, compared to accelerometers. However, the study of Oliver et al. (2007) indicated that pedometers are suitable for situations where a general idea of preschoolers’ PA is required, which was the aim of the current study [32]. Furthermore, results on the country-specific level should be interpreted cautiously because in some of the countries (i.e., Bulgaria, Germany and Greece) data originate from only one city. Due to the recruitment of preschoolers in selected areas within each country, the ToyBox-sample is not a fully representative European sample. However, within the ToyBox-sample preschoolers were included from different socio-economic backgrounds (low, medium and high), and in each kindergarten (almost) complete classes were included. Thus, the sample can give a fair approximation of the current situation within Europe. Besides, as already mentioned before, preschoolers were recruited through kindergartens, daycare centers and preschools. However, data regarding the frequencies in each group or how these groups were represented across countries have not been recorded in the ToyBox-study. Finally, all measurements were collected between May and June 2012 across the participating countries, which strengthens the comparability across the countries. However, an overestimation of preschoolers’ step counts may have occurred since the colder months (e.g., December, January) were not part of the measurement timeframe. Due to health promotion initiatives in European countries in recent years, PA data, based on data collected in 2012, may slightly differ from the current situation (2018).

There are also some strengths that should be acknowledged. First, a large sample size of 3578 preschoolers was included in this study, although we used a strict number of included valid days (i.e., two valid weekdays and two valid weekend days). Additionally, step count patterns were described across six different countries, which made it possible to compare cross-European step count patterns during weekdays and weekend days. Finally, multilevel analyses were used to take clustering of preschoolers at five levels into account.

## 5. Conclusions

Preschoolers’ step count patterns were described and compared across six European countries taking into account the daily step counts during weekdays and weekend days. Weekday step count patterns were related to daily kindergarten schedules across all countries. It became clear that differences in step count patterns were observed across the countries both during weekdays and weekend days, especially between Southern and Central European countries, which may be explained by differences in (school) policy, lifestyle habits or culture. During weekdays, special attention is needed to enhance preschoolers’ PA levels during after school hours across the Central European countries. Furthermore, in particular in Bulgaria and Greece, a recommendation might be to avoid putting all preschoolers in bed in the afternoon, but to respond to the needs of each individual preschoolers instead, as low step counts in preschoolers may be partly explained by the afternoon sleep period. During weekend days, preschoolers in Central European countries should be stimulated to be more physically active during the afternoon. On the other hand, taking into account the common habit of taking a siesta in Southern European countries, preschoolers can be encouraged to enhance their PA levels in the morning and in the late afternoon or evening. It is important to take these step count patterns into account in the development of interventions to promote PA in preschoolers.

## Figures and Tables

**Table 1:** Descriptive statistics of preschoolers across the participating European countries.

Country	*n*	Mean Age in Years (±SD)	Boys (%)	Low Educational Level Mother (%) (≤14 Years of School Education)
Belgium	642	4.4 (±0.5)	54.1	33.4
Bulgaria	446	4.9 (±0.3)	47.8	41.3
Germany	396	4.5 (±0.6)	51.0	54.0
Greece	515	4.9 (±0.3)	50.9	49.7
Poland	1069	4.9 (±0.3)	53.6	21.0
Spain	510	4.9 (±0.3)	53.9	35.6
Total	3578	4.8 (±0.4)	52.3	35.5

**Table 2:** Amount of step counts in preschool boys and girls, across the participating countries.

	Gender	N	Average Number of Step Counts (±SD)
Weekdays	Boys	1870	11,169 (±3364) *
	Girls	1704	9970 (±3001) *
Weekend days	Boys	1829	10,164 (±3894) *
	Girls	1661	9288 (±3549) *

* Significant gender differences *p* < 0.05.

**Table 3:** Number of steps in significant peaks and troughs during weekdays.

	Belgium	Bulgaria	Germany	Greece	Poland	Spain
**Peak 1**	931 ^+^	734 ^+^	1065 ^++^	632 ^+^	697 ^+^	845 ^+^
**Trough 1**	681 ^−^	542 ^−^	860 ^− −^	446 ^−^	523 ^−^	640 ^−^
**Peak 2**	1104 ^$^	940 ^++^	1089 ^+++^	1090 ^++^	1188 ^++^	1178 ^++^
**Trough 2**	791 ^$^	145 ^− −^	-	529 ^− −^	515 ^− −^	670 ^− −^
**Peak 3**	1154 ^++^	1360 ^+++^	-	845 ^+++^	1112 ^+++^	1224 ^+++^

-: No significant peak or trough/no significant increase or decrease; ^+^: General peak 1; ^++^: General peak 2; ^+++^: General peak 3; ^−^ : General trough 1; ^− −^: General trough 2; ^$^: peak or trough in European country.

**Table 4:** Number of steps in significant peaks 1, 2 and in significant trough 1 during weekend days.

	Belgium	Bulgaria	Germany	Greece	Poland	Spain
**Peak 1**	-	941	993	842	1022	1119
**Trough**	-	459	807	417	933	473
**Peak 2**	-	961	990	909	1032	1093

## Supplementary Materials

The following are available online at http://www.mdpi.com/1660-4601/15/2/291/s1, File 1: Description of daily kindergarten schedules in six European countries; File 2: Preschoolers’ PA levels in each country.

## Availability of Data and Material

The datasets analyzed during the current study are available from the corresponding author on reasonable request.

## Abbreviations

PA	Physical activity
SES	Socio-economic status
