# Supplementary material for: Daily Patterns of Preschoolers’ Objectively Measured Step Counts in Six European Countries: Cross-Sectional Results from the ToyBox-Study
Source: Int J Environ Res Public Health. 2018 Feb 7;15(2):291. doi: 10.3390/ijerph15020291 (PMC5858360; doi:10.3390/ijerph15020291)
Supplement: Supplementary file 1 [file ijerph-15-00291-s001.zip › Additional file 2.pdf]

	**Belgium** n = 806	**Bulgaria** n = 470	**Germany** n = 449	**Greece** n = 575	**Poland** n = 1,192	**Spain** n = 553
Meeting PA-guidelines[1] (%)						
Weekday	40.0 [c,d,e,g]	29.3 [b,d,f,g]	49.9 [a]	26.5 [,d,f,g]	43.2 [c,d,e,g]	60.7 [a]
Weekend day	20.5 [c,d,f,g]	29.2 [b,e,f,g]	31.4 [b,e,f,g]	20.3 [c,d,f,g]	41.8 [a]	37.0 [a]

PA = Physical Activity; SE = Standard Error;

[a] significantly different from the other countries;

[b] significantly different Belgium;

[c] significantly different from Bulgaria;

[d] significantly different from Germany;

[e] significantly different from Greece;

[f] significantly different from Poland;

[g] significantly different from Spain;

[1] _ 11,500 steps per day
