# Supplementary material for: Daily Patterns of Preschoolers’ Objectively Measured Step Counts in Six European Countries: Cross-Sectional Results from the ToyBox-Study
Source: Int J Environ Res Public Health. 2018 Feb 7;15(2):291. doi: 10.3390/ijerph15020291 (PMC5858360; doi:10.3390/ijerph15020291)
Supplement: Supplementary file 1 [file ijerph-15-00291-s001.zip › Additional file 1.pdf]

Belgium

08 AM - 08:30 AM	Start preschool
08:30 AM - 10 AM	Classroom activities
10 AM - 10:30 AM	Morning recess
10:30 AM - 12 PM	Classroom activities
12 PM - 1:30 PM	Lunch break+ recess
1:30 PM - 2:30 PM	Afternoon recess
2:30 PM - 3:30 PM	Classroom activities
3:30 PM - 4:30 PM	End hour preschool

Bulgaria

8 AM - 9 AM	Start kindergarten
9 AM - 10 AM	Breakfast
10 AM - 11 AM	Classroom activities, midmorning fruit snack
11 AM - 12 PM	Recess
12 PM - 1 PM	Lunch break
1 PM - 4 PM	Afternoon sleep / Free play time
4 PM - 5:30 PM	End hour kindergarten

Germany (Differs across preschools, example of one kindergarten)

7 AM - 8 AM	Start kindergarten
8 AM - 9 AM	Breakfast
9 AM - 11 AM	Free play time/Classroom activities
11 AM - 12 PM	Recess
12 PM - 1 PM	Lunch break
1:30 PM - 2:30 PM	Afternoon sleep period, classroom activities (quiet play activities)

2:30 PM - 3:30 PM	Free play time
3:30 PM - 4:00 PM	Snacktime
4:00 PM - 5:30 PM	Playtime/End hour kindergarten

Greece

8 AM - 9 AM	Start childcare centers and preschools
9 AM - 10:30 AM	Classroom activities
10:30 AM - 11:15 AM	Break to eat and recess
11:45 AM - 12:15 PM	Prepare to leave from kindergarten
1:45 PM - 2:30 PM	Afternoon sleep period
2:30 PM - 3:15 PM	Classroom activities
3:45 PM - 4 PM	End hour childcare centers and preschools.

Poland

8 AM - 9 AM	Start kindergarten
9 AM - 11 AM	Classroom activities
11 AM - 12 PM	Recess
12 PM - 1 PM	Lunch break
1 PM - 2 PM	Afternoon sleep
2 PM - 3 PM	Free play time
3 PM - 4 PM	End hour kindergarten

Spain

8 AM - 9 AM	Start preschools
9 AM - 10 AM	Classroom activities (quiet play activities)
10 AM - 11 AM	Break
11 AM - 1 PM	Classroom activities

1 PM - 3 PM	Lunch + recess
3 PM - 4:30 PM	Classroom activities (quiet play activities)
4:30 PM - 5 PM	End hour preschool (preschoolers go home or participate in extracurricular activities)
